# Supplementary material for: Correlation of Computed Tomography Parameters with Histology, Stage and Prognosis in Surgically Treated Thymomas
Source: Medicina (Kaunas). 2020 Dec 24;57(1):10. doi: 10.3390/medicina57010010 (PMC7824084; doi:10.3390/medicina57010010)
Supplement: Supplementary file 1 [file medicina-57-00010-s001.zip › Supplementary Table S1.pdf]

Supplementary Table 1 - CT scan features

**Total No.**	**50**
Location	
Right	13 (26%)
Left	27 (54%)
Median	10 (20%)
Size (± SD; range)	
Major axis (mm)	59 ± 26.9; 21-132
Minor axis (mm)	38.7 ± 20.5; 10-94
Volume (ml)	114.9 ± 176.9; 3-994
Regular shape	30 (60%)
Regular contours	19 (38%)
Necrosis	24 (48%)
Calcifications	13 (26%)
Lymphadenopathy	10 (20%)
Pleural	
Contact length (± SD; range, mm)	68.2 ± 34.8; 15-175
Effusion	4 (8%)
Dissemination	2 (4%)
Pericardial effusion	5 (10%)
Invasion	
Mediastinal fat	23 (46%)
Major blood vessels	44 (88%)
Pericardium	40 (80%)
Lung	2 (4%)
Homogenous contrast enhancement pattern	11 (32% over 34 patients)
