# Supplementary material for: Correlation of Computed Tomography Parameters with Histology, Stage and Prognosis in Surgically Treated Thymomas
Source: Medicina (Kaunas). 2020 Dec 24;57(1):10. doi: 10.3390/medicina57010010 (PMC7824084; doi:10.3390/medicina57010010)
Supplement: Supplementary file 1 [file medicina-57-00010-s001.zip › Supplementary Table S2.pdf]

Supplementary Table 2 -Correlation of CT parameters and histological classifications

		WHO						SUSTER AND MORAN		
		A	AB	B1	B2	B3	p	Well-differentiated	Moderately-differentiated	p
Long axis (median, mm)	< 53.5	3 (75%)	6 (42.9%)	5 (45.5%)	3 (37.5%)	8 (61.5%)	0.63	17 (45.9%)	8 (61.5%)	0.33
	> 53.5	1 (25%)	8 (57.1%)	6 (55.5%)	5 (62.5%)	5 (38.5%)		20 (54.1%)	5 (38.5%)	
Short axis (median, mm)	< 36.5	2 (50%)	7 (50%)	6 (54.5%)	3 (37.5%)	7 (53.8%)	0.95	18 (48.6%)	7 (53.8%)	0.75
	> 36.5	2 (50%)	7 (50%)	5 (45.5%)	5 (62.5%)	6 (46.2%)		19 (51.4%)	6 (46.2%)	
Volume (median, mm^3)	< 60	3 (75%)	7 (50%)	6 (54.5%)	2 (25%)	7 (53.8%)	0.53	18 (48.6%)	7 (53.8%)	0.75
	> 60	1 (25%)	7 (50%)	5 (45.5%)	6 (75%)	6 (46.2%)		19 (51.4%)	6 (46.2%)	
Length of pleural contact (median, mm)	< 64	4 (100%)	7 (50%)	5 (45.5%)	2 (25%)	7 (53.8%)	0.19	18 (48.6%)	7 (53.8%)	0.75
	> 64	0	7 (50%)	6 (55.5%)	6 (75%)	6 (46.2%)		19 (51.4%)	6 (46.2%)	
Shape	Oval	3 (75%)	13 (92.9%)	6 (54.5%)	5 (62.5%)	3 (23.1%)	**0.007***	27 (73%)	3 (23.1)	**0.002***
	Irregular	1 (25%)	1 (7.1%)	5 (45.5%)	3 (37.5%)	10 (76.9%)		10 (27%)	10 (76.9%)	
Contours	Regular	2 (50%)	9 (64.3%)	4 (36.4%)	4 (50%)	0	**0.012***	19 (51.4%)	0	**0.001***
	Irregular	2 (50%)	5 (35.7%)	7 (63.6%)	4 (50%)	13 (100%)		18 (48.6%)	13 (100%)	
Necrosis	Yes	2 (50%)	6 (42.9%)	6 (54.5%)	4 (50%)	6 (46.2%)	0.98	18 (48.6%)	6 (46.2%)	0.88
	No	2 (50%)	8 (57.1%)	5 (45.5%)	4 (50%)	7 (53.8%)		19 (51.4%)	7 (53.8%)	
Calcifications	Yes	2 (50%)	3 (21.4%)	0	3 (37.5%)	5 (38.5%)	0.145	8 (21.6%)	5 (38.5%)	0.23
	No	2 (50%)	11 (78.6%)	11 (100%)	5 (62.5%)	8 (61.5%)		29 (78.4%)	8 (61.5%)	
Lymph node enlargements	Yes	1 (25%)	2 (14.3%)	2 (18.2%)	3 (37.5%)	2 (15.4%)	0.77	8 (21.6%)	2 (15.4%)	0.22
	No	3 (75%)	12 (85.7%)	9 (81.9%)	5 (62.5%)	11 (84.6%)		29 (78.4%)	11 (84.6%)	
Pleural effusion	Yes	1 (25%)	2 (14.3%)	0	1 (12.5%)	0	0.33	4 (10.6%)	0	0.22
	No	3 (75%)	12 (85.7%)	11 (100%)	7 (87.5%)	13 (100%)		33 (89.2%)	13 (100%)	
Pericardial effusion	Yes	1 (25%)	2 (14.3%)	0	0	2 (15.4%)	0.43	3 (8.1%)	2 (15.4%)	0.45
	No	3 (75%)	12 (85.7%)	11 (100%)	8 (100%)	11 (84.6%)		34 (91.9%)	11 (84.6%)	
Invasion of mediastinal fat	Yes	1 (25%)	3 (21.4%)	6 (54.5%)	4 (50%)	9 (69.2%)	0.12	14 (37.8%)	9 (69.2%)	0.051
	No	3 (75%)	11 (78.6%)	5 (45.5%)	4 (50%)	4 (30.8%)		23 (62.2%)	4 (30.8%)	
Contact with mediastinal vessels	Yes	3 (75%)	11 (78.6%)	10 (90.9%)	7 (87.5%)	13 (100%)	0.45	31 (83.6%)	13 (100%)	0.12
	No	1 (25%)	3 (21.4%)	1 (9.1%)	1 (12.5%)	0		6 (16.2%)	0	
Pericardial contact	Yes	2 (50%)	10 (71.4%)	9 (81.8%)	6 (75%)	13 (100%)	0.18	27 (73%)	13 (100%)	**0.036***
	No	2 (50%)	4 (28.6%)	2 (18.2%)	2 (25%)	0		10 (27%)	0	
Lung invasion	Yes	0	1 (7.1%)	0	0	1 (7.7%)	0.78	1 (2.7%)	1 (7.7%)	0.43
	No	4 (100%)	13 (92.9%)	11 (100%)	8 (100%)	12 (92.3%)		36 (97.3%)	12 (92.3%)	
Pleural invasion	Yes	1 (25%)	1 (7.1%)	0	0	0	0.18	2 (5.4%)	0	0.39
	No	3 (75%)	13 (92.9%)	11 (100%)	8 (100%)	13 (100%)		35 (94.6%)	13 (100%)	
Contrast enhancement diffusion pattern	Homogeneous	2 (66.7%)	2 (25%)	3 (37.5%)	1 (16.7%)	3 (33.3%)	0.63	8 (32%)	3 (33.3%)	0.94
	Inhomogeneous	1 (33.3%)	6 (75%)	5 (62.5%)	5 (83.3%)	6 (66.7%)		17 (68%)	6 (66.%)	
Laterality	Right	0	4 (28.6%)	4 (36.4%)	2 (25%)	3 (23.1%)	0.07	10 (27%)	3 (23.1%)	**0.02***
	Left	4 (100%)	9 (64.3%)	4 (36.4%)	6 (75%)	4 (30.8%)		23 (62.2%)	4 (30.8%)	
	Median	0	1 (7.1%)	3 (27.3%)	0	6 (46.2%)		4 (10.8%)	6 (46.2%)	
